# Oviposition by Female *Plodia interpunctella* (Lepidoptera: Pyralidae): Description and Time Budget Analysis of Behaviors in Laboratory Studies

**DOI:** 10.3390/insects7010004

**Published:** 2016-01-22

**Authors:** Kishan R. Sambaraju, Sarah L. Donelson, Janko Bozic, Thomas W. Phillips

**Affiliations:** 1 Department of Entomology and Plant Pathology, Oklahoma State University, Stillwater, OK 74078, USA; sarah.l.donelson@monsanto.com (S.L.D.); twp1@ksu.edu (T.W.P.); 2 Natural Resources Canada, Canadian Forest Service, 1055 du PEPS, Québec, QC G1V 4C7, Canada; 3 Monsanto Company, 800 North Lindbergh Blvd., St. Louis, MO 63167, USA; 4 Department of Biology, University of Ljubljana, Ljubljana 1000, Slovenia; Janko.Bozic@bf.uni-lj.si; 5 Department of Entomology, 123 W. Waters Hall, Kansas State University, Manhattan, KS 66506, USA

**Keywords:** *Plodia interpunctella*, Indian meal moth, oviposition, behavior, scotophase, photophase, stored products

## Abstract

The oviposition behavior of the Indian meal moth, *Plodia interpunctella* (Hübner) (Lepidoptera: Pyralidae), a major insect pest of durable stored foods, was studied in small experimental arenas under laboratory conditions using videography, and a time budget analysis of its behaviors was documented. Resting gravid females typically became active shortly after the start of the scotophase. The characteristic behaviors exhibited by mated females prior to oviposition included antennal movement, grooming of antennae and mouth parts using the forelegs, walking or flying, and abdomen bending and dragging. Pre-oviposition behaviors such as antennal grooming and walking or flying were observed to alternate several times before females commenced the abdominal dragging behavior that preceded egg laying. Eggs were laid singly or sometimes in groups, either freely or stuck to food material. Gravid females showed little or no movement during the photophase; however, they actively flew and oviposited during the scotophase. Females allocated only a small portion of their time to oviposition while the rest of the time was spent away from food. Females oviposited on food material by making repeated visits, predominantly during the first four hours of the scotophase. Visits and time spent on food declined as the scotophase advanced.

## 1. Introduction

The Indian meal moth, *Plodia interpunctella* (Hübner) (Lepidoptera: Pyralidae), is a worldwide pest of stored and processed durable food commodities [1]. Adult populations are generally found in high numbers in or near flour mills [2], food warehouses [3,4], and retail stores [5]. This short-lived insect (7–10 days) is nocturnal and becomes reproductively active within 24 h of eclosion from pupae. Mating occurs when “calling” or pheromone-releasing females induce males to copulate in a characteristic sequence of events [6,7]. Mated females lay 100–150 eggs on average during their lifetime, depending on the quality of the food. Larvae hatch from the eggs in 3–4 days under ambient conditions. Economic losses may occur due to the degraded food quality resulting from larval feeding and webbing that make the food unfit for human consumption, or due to the presence of high numbers of live or dead adults in bulk storages, which may result in downgrading of the stored grain [8,9].

Biotic and abiotic factors influence host selection and oviposition by the female *P. interpunctella*. For example, conspecific [10] and host-related factors [11,12,13,14,15] play key roles in eliciting upwind flight, landing, and egg-laying behaviors from gravid females. Optimal ovipositional response is elicited only when a female comes into contact with the host surface [16]. At this stage, physical and chemical stimuli on the substrate surface play a very important role [17]. Females prefer to oviposit on substrates that offer an appropriate combination of chemical and physical cues, while substrates lacking these cues may be rejected. Host extract-coated spherical substrates with a smooth exterior surface are preferred for oviposition compared to similarly-coated flat, rough surfaces [17]. Although host selection is relatively well understood in *P. interpunctella*, information gaps exist in our understanding of specific behaviors of individual moths associated with the act of oviposition itself. For example, ovipositing females show interesting behaviors such as sticking their eggs directly on seed-resembling artificial substrates and/or laying the eggs through the interstitial gaps among them that were observed in a previous work by some of us [17]. Descriptive accounts of ovipositional behaviors, time allocation by gravid females prior to, during, and after oviposition, and visit patterns toward a food source by ovipositing *P. interpunctella* females have been sparsely documented [9], if at all, to our knowledge. This paper intends to fill these knowledge gaps in the ovipositional behavior of *P. interpunctella*. Specifically, the objectives of this laboratory study were to (1) describe and document the behavioral sequences performed by gravid females leading up to oviposition; (2) analyze the time budget/allocation of individual ovipositing females using an automated animal tracking system over a 48-h period; and (3) analyze the visit patterns of females toward a food source used for oviposition.

## 2. Experimental Section

### 2.1. Insects

Adult insects used in our studies were obtained from a laboratory colony of moths reared on a cornmeal-based diet [10] at 28 °C, 60%–70% r.h., and under 16L:8D photoperiod conditions. Cardboard rolls (2–2.5 cm wide) were added to the culture jars (0.47 L) when the wandering-stage, fifth-instar larvae were observed. These larvae eventually pupated in the cardboard rolls. The rolls were removed after one week, pupae were separated by sex and placed individually into 4.5-cm glass shell vials in a growth chamber under conditions identical to those mentioned above. Adults emerging from these pupae were used in the behavioral experiments.

### 2.2. Study 1: Behaviors Exhibited by Female P. interpunctella during Oviposition

The oviposition behavior of *P. interpunctella* females was observed in a custom-made prefabricated Plexiglas arena (34 cm × 29 cm × 5.5 cm). The side walls of the arena slanted outward at an obtuse angle to the floor of the arena. The inside surface of the arena was covered with a sheet of white paper so as to provide bright contrast against the dark moth body. This set-up made it possible to observe behaviors (e.g., antennal movement) when the moth was on the walls of the arena, and it also provided a reflective surface for incident light that enhanced overall visibility inside the arena for recording purposes. A fresh sheet of white paper was used for each trial. Two 9-cm diameter plastic Petri dish bottoms were placed 5 cm apart on the floor of the arena, one of which was empty while the other contained 3 g of whole soybean, *Glycine max* (L.) (Fabaceae), and 1 g of cracked soybean. The empty dish was included because *P. interpunctella* females, in some instances, lay eggs when just the thigmotactic stimuli (which in this case were offered by the dish edges) are present. Cracked soybeans that passed through a US #10 sieve and that were collected on a US #14 sieve (Seedburo Equipment Co., Chicago, IL, USA) were used for the experiment. A separate square-shaped piece of dark transparency film was placed underneath each dish on the floor of the arena. *Plodia interpunctella* eggs are small (~0.5 mm), oval, and milky white in color [18]. Hence, the presence of a dark background underneath each dish made it easier to capture an act of egg deposition by a female moth. Direct lighting as well as light reflected off the white paper facilitated observations of oviposition behaviors within the dish. Soybean was used because an earlier study showed that soybean was a good larval food source that elicited an optimal ovipositional response from adult *P. interpunctella* females [15].

A virgin female (<2 days old) was paired inside a 4.5-cm glass vial with a male moth of the same age for 24 h to allow copulation prior to the start of the experiment. Care was taken to make sure that each female used in the study was presumably mated by selecting females from copulating pairs. Eighty five to 100% of *P. interpunctella* females, ≤3 days old, when mated with male moths of the same age, will have at least one spermatophore in their bursa copulatrix, which indicates that the mating was successful [19]. Light-dark settings in the growth chamber housing the moth colony were adjusted to allow scotophase (or “dark phase”) observations during regular work hours without disturbing the moths’ circadian rhythm. A single mated female was released into the experimental arena 15 min before the start of the scotophase, and a transparent Plexiglas sheet was used to cover the top portion of the arena to prevent the moth from escaping. Scotophase illumination for this experiment was provided by two 12-per-strip (30.1 cm × 2.1 cm) red light-emitting diodes (LEDs; λ = 630 nm; The LED Light Inc., Carson City, NV, USA). The LED strips were clamped to a ring stand and positioned above the arena. The distance between the stand and the arena (~70 cm) was adjusted such that light intensity at the level of the arena was low enough for the moths to exhibit oviposition behaviors, yet high enough to allow video recording. Ambient environmental conditions in the experimental room were in the range of 24–25 °C and 28%–32% r.h. Insect behaviors were recorded on a digital tape using a video recorder (Elura 100, Canon USA, Inc., Melville, NY, USA) from the start of the scotophase. Direct visual observations of moth behaviors were also made. An experiment ended when the female successfully oviposited and left the food dish. Eggs in the dishes and in the arena proper (*i.e.*, outside the dishes) were counted at the end of the experimental period. Moths that failed to become active within 30 min from the start of the scotophase, or that did become active but did not oviposit within 60 min, were not used in our analyses of oviposition behavior. Nevertheless, the latter type of moths displayed identical pre-ovipositional behaviors compared to moths that eventually oviposited. Illustrations of the different behavioral events that culminated in oviposition were made based on video recordings of successfully ovipositing moths (*n* = 9 individual moths that completed oviposition).

### 2.3. Study 2: Analysis of P. interpunctella Time Allocation Behavior during Oviposition

Time allocation behavior of gravid *P. interpunctella* females among three potential oviposition sites was studied over a 48-h period using Viewer animal tracking system (Biobserve GmbH, Bonn, Germany). The study was conducted under ambient environmental conditions of 24–25 °C, 30%–40% r.h. and L16:D8 photoperiod. A Plexiglas arena identical to that used for Study 1, with its inside surface covered with white paper, was used in this study. A fresh sheet of white paper was used in each trial. The arena contained two 3.5-cm diameter plastic Petri dish bottoms that were placed approximately 5–6 cm apart. The food dish contained 2 g of cracked soybean that was evenly spread on the bottom surface of the Petri dish in order to avoid dark areas that would otherwise interfere with the automated recording of the moth on the food during the scotophase. An empty dish served as a control. Automated recording of insect movements using an animal tracking system is based on the contrast of the insect with its surroundings. In other words, an insect is recognized as a dark object on a bright background. Only cracked soybean was used in this study because it provided a good background for tracking the female moth when it moved over the food during oviposition. The video capture device was a Sony SSC-M183 B&W analog camera that was raised to a height of 1 m above the arena using a tripod. The camera was connected to a computer via an analog-to-digital video converter (Canopus ADVC55 Advanced DV Converter, Canopus Corporation, San Jose, CA, USA). Photophase (“light phase”) illumination was provided by two overhead 34 W fluorescent light bulbs (Ecologic^®^; Sylvania Company, Danvers, MA, USA). Light during the scotophase was provided by a combination of low-intensity red and infrared lights (Kemo-Electronic GmbH, Geestland, Germany). Mated females were obtained as described in Study 1. A single mated female was released in the arena and a transparent Plexiglas sheet was used to cover the top of the arena in order to prevent the moth from escaping. Then, the video tracking system was started to record the movements of the moth over a 48-h period. We present data for: (a) the proportion of time spent inside or on the outside surface of the food or empty dish, and time spent in the arena proper during the light and dark phases over the 48-h observation period; and (b) the number of eggs laid in the dishes and in the arena proper (*n* = 6 individual moths, each tracked for 48 h).

### 2.4. Study 3: Real-Time Analysis of the Visit Pattern of P. interpunctella Females toward a Food Source

Video tracking of *P. interpunctella* females indicated that the females made several visits to the food dish during the scotophase. To confirm this finding, we studied the visit patterns of *P. interpunctella* females toward a food source in 15-cm diameter glass Petri dishes that housed a single 3.5-cm diameter plastic Petri dish bottom containing 1 g of cracked soybean. The smaller Petri dish was placed on a square-shaped dark transparency background to observe eventual egg laying events. Temperature and relative humidity in the test room were in the range of 24–25 °C and 24%–32% r.h., respectively. A single mated female (~2 days old) was released into the experimental arena 15 min before the start of recording. Similar to Study 2, an analog video camera was set approximately 1.2 m above the arena and connected to a computer through an analog-to-digital video converter. The video was recorded directly on a local hard drive using Windows Movie Maker^®^ software application (Microsoft Corp., Redmond, WA, USA). Video recording began 1 h before the start of the scotophase, which lasted for 8 h. Video recording during the scotophase was facilitated by the use of red LEDs. Recording was stopped 1 h into the photophase that followed the 8-h scotophase. A total of 20 h of video was recorded (10 h each for “day 1” and “day 2”) for each female that was confined to the experimental arena for a 48-h period; four separate moths were observed in this study. Data were recorded for the total number of visits per female during each of the two consecutive 10-h periods (in total, 80 h of video data), time spent by the moth inside or on the outside surface of the food dish during each visit, latency time between visits, and the number of eggs laid.

### 2.5. Data Analyses

Data obtained for egg counts in the food dish, in the empty dish, and in the arena proper from Study 1 and Study 2 were analyzed using a Chi-square test to examine whether distribution of the eggs in the three oviposition sites significantly departed from what would be expected if eggs were laid randomly. When significant differences were observed, a series of paired *t*-tests were conducted to assess whether pairwise differences existed in the log-transformed [log_10_ (x + 1)] egg counts among the food dish, the empty dish, and the arena proper using SAS software v. 9.3 [20]. We studied the association between the time spent by *P. interpunctella* females by phase (two levels: photophase and scotophase) and the site of oviposition (three levels: food dish, empty dish and the arena proper) in Study 2 in a 2 × 3 contingency table, and a test of independence between the two variables was performed using a Chi-square test. Then, paired *t*-tests were conducted, both within a phase among the three oviposition sites, and between the two phases for each oviposition site, after converting the time spent at each of the sites to arcsine-transformed proportions [21] of total time per phase. In Study 3, the number of visits and the total time spent by the females on the food dish on day 1 *versus* day 2 were analyzed by paired *t*-tests. To account for multiple pairwise comparisons, a conservative *P*-level of 0.01 was chosen to assess the significance of the tests. Finally, a graphical representation of time spent by female moths on the food dish in Study 3 was done by averaging the time spent on the food every 15 min starting from 00:00 to 10:00 h for the four replicates of data. Only untransformed data are presented in the figures and tables.

## 3. Results

### 3.1. Study 1: Behaviors Exhibited by P. interpunctella Females during Oviposition

**Figure 1:**
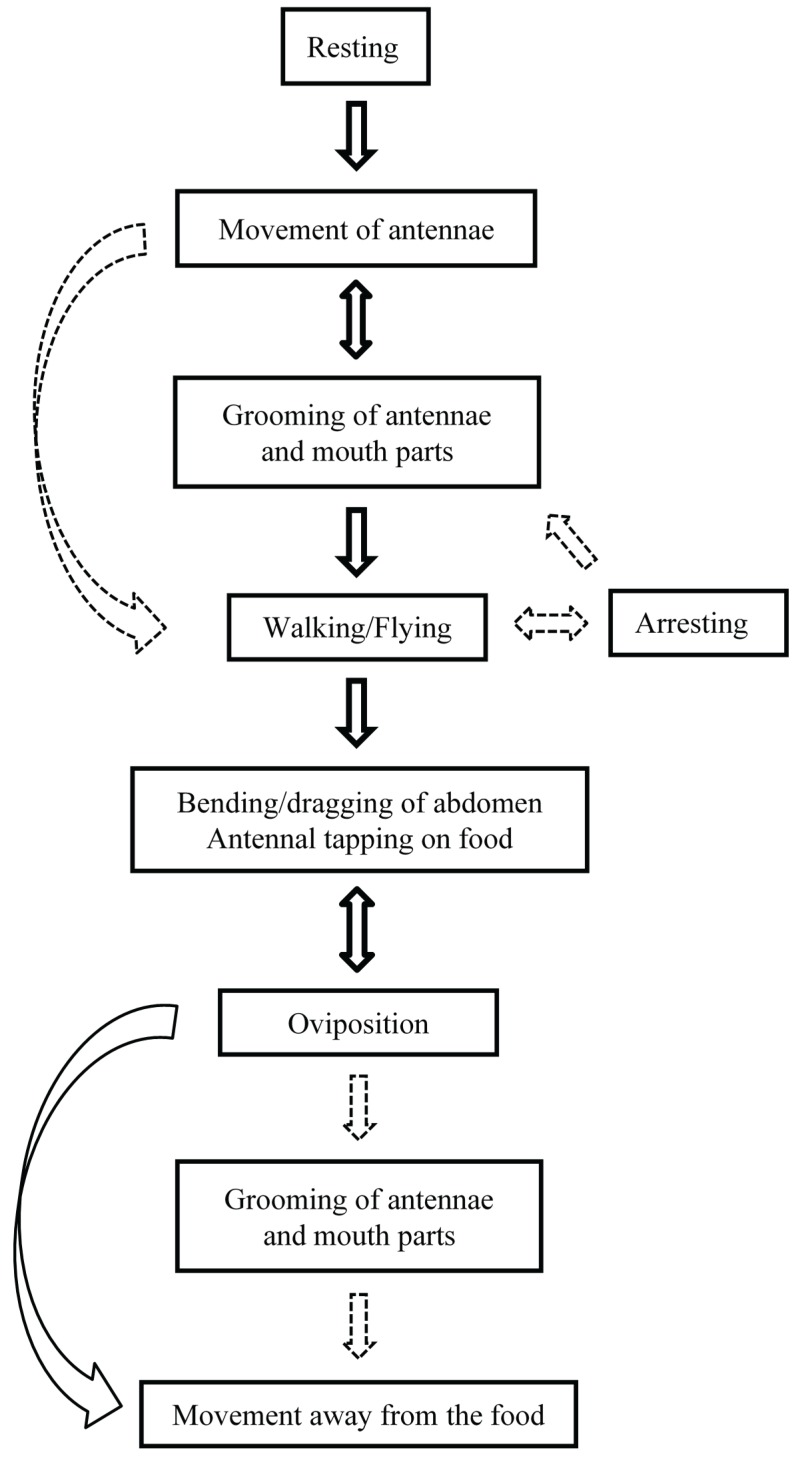
A representative diagram of sequential pre- and post-oviposition behaviors displayed by gravid female *Plodia interpunctella*. Solid arrows indicate frequently observed behaviors, whereas dashed arrows indicate occasional behaviors. Bidirectional arrows indicate behaviors that occur alternatingly with another behavior.

**Figure 2:**
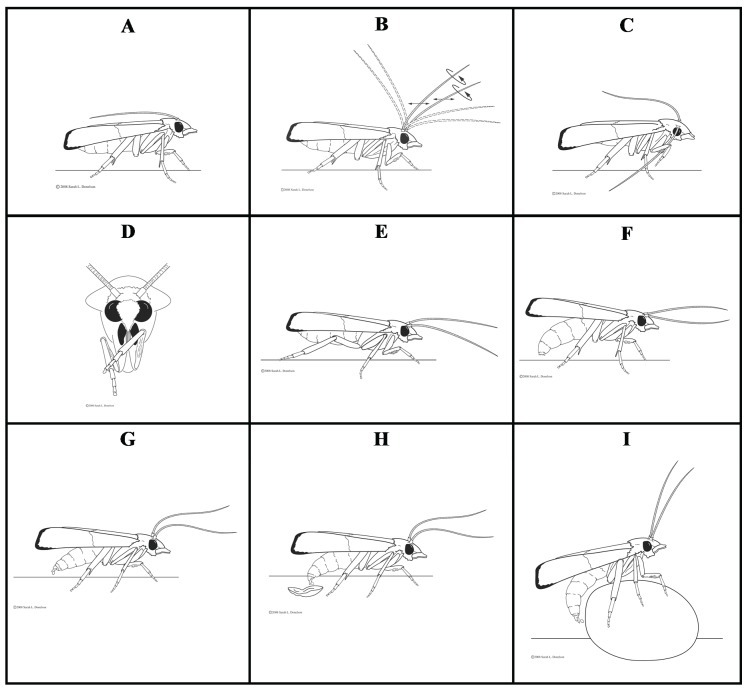
A generalized depiction of the sequence of behaviors displayed by gravid *Plodia interpunctella* females before and during oviposition that include (**A**) resting; (**B**) antennal movement; (**C**) grooming of antenna; (**D**) grooming of mouthparts; (**E**) walking; (**F**) abdomen bending and dragging; and (**G**–**I**) oviposition.

After the transition from a photophase to a scotophase, gravid *P. interpunctella* females displayed several prominent behaviors leading up to oviposition. These included: (1) resting; (2) antennal movement; (3) grooming of antennae and mouth parts; (4) walking or flying; (5) abdomen bending and dragging; and (6) oviposition (Figure 1 and Figure 2A–I). A description of these behaviors follows.

#### 3.1.1. Resting

Female moths “rested” with their antennae folded on the back of their body, close to the wings, at the start of the scotophase (Figure 2A). Legs were kept close to the body and the head was in a slightly raised posture such that the insect appeared compact.

#### 3.1.2. Antennal Movement

Gravid females generally became active within 5–10 min of the start of the scotophase. The antennae were either raised in a slow, gradual manner or in an abrupt fashion. The antennae were then moved in a counter-clockwise direction in an elliptical pattern with greater eccentricity, *i.e.*, in progressively larger ellipses. Initially, the movement was slow, but it became faster as the behavior proceeded. The female moths then showed a mix of opposing and unidirectional movements of the right and the left antenna and the increasing frequency of these movements caused a reduction in the angle of divergence between the antennae. As the moths became increasingly active, the eccentric antennal movement turned more linear and the two antennae moved almost parallel to each other (Figure 2B).

#### 3.1.3. Grooming

**Figure 3:**
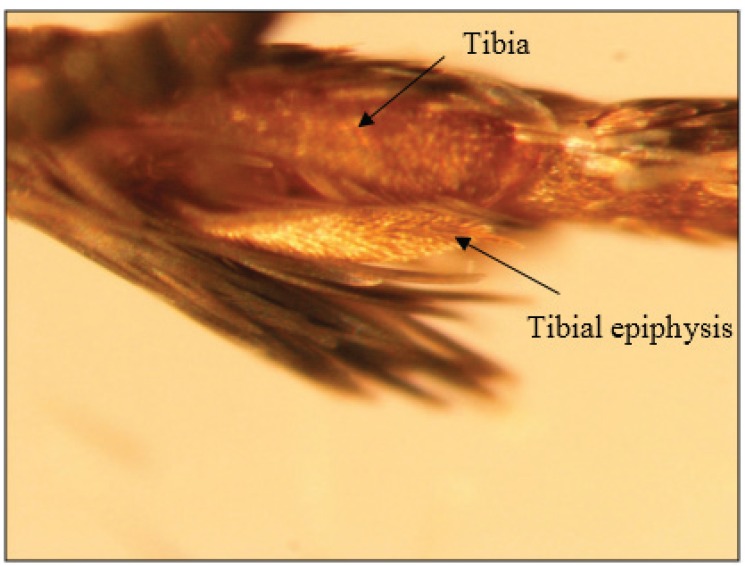
Image of the brush-like tibial epiphysis on the fore tibia of *Plodia interpunctella* females that may be involved in grooming the antennae during oviposition. The image was recorded using an Olympus SZX stereo microscope (Olympus America Inc., Center Valley, PA, USA) at 115× magnification. Note that the scales on the fore tibia were removed before the image was taken.

Active moths “groomed” their antennae and mouth parts with their fore legs. The antennae were lowered toward the substrate surface along the plane of the fore legs. Then, a single fore leg was raised and the antenna on the same side was slid through an area close to the junction of the fore tibia with the fore tarsi (Figure 2C). Similarly, the other antenna was groomed with the foreleg of its corresponding side. A brush-like “tibial epiphysis” on the fore tibia was apparently involved in grooming the antennae (Figure 3). Labial palpi and the proboscis were also groomed in tandem with the antennae (Figure 2D). Gravid females were observed to groom their antennae and mouth parts one to several times in the manner described above; only in rare cases did they skip this behavior.

#### 3.1.4. Walking and Flying

Females were observed walking or flying after a certain period of antennal movement and grooming. When a mated female walked, her antennae were positioned anterior to the body in the direction of taxis and close to the substrate (Figure 2E). Occasional tapping of the substrate surface with the antennae was observed during walking. In some cases, a female paused (“arresting” behavior) several times and then resumed walking, or she would take flight. During those short intermittent stops, the moths sometimes groomed their antennae and mouth parts, and then resumed walking or took flight.

#### 3.1.5. Abdomen Bending and Dragging

During walking or immediately after encountering the oviposition resource, females were observed bending their posterior (*i.e.*, distal) abdominal segments toward the substrate and then dragging their abdomen (Figure 2F). The ovipositor was then seen touching the substrate surface in a back-and-forth motion during this abdomen-dragging behavior. Females were observed probing their ovipositors on the broken food particles by moving the abdomen laterally from one side to the other. Also, the females apparently perceived the curvature of the whole bean by climbing on it and aligning their abdomen along the curvature of the bean (Figure 2F). This behavior occurred concurrently with periodic antennal tapping on the food. The females walked around the food, paused at several places, probed the food with their bent abdomen, and then proceeded to oviposit on or near the bean.

#### 3.1.6. Oviposition

*Plodia interpunctella* females oviposited only in the food dish, and not in the empty dish, through a back and forth movement of the ovipositor. Moths were observed to lay eggs freely on a substrate near the food, or stick their eggs on cracked food material. When eggs were laid freely, they appeared to be “squirted” out from the ovipositor one at a time with the female maintaining a relatively straight abdomen (Figure 2G). Occasionally, a single egg or a concatenate of 2–4 eggs were laid in succession and appeared to be glued to the food surface (Figure 2H). Oviposition was accomplished by a series of abdominal contractions that resulted in the expulsion of an egg from the ovipositor. After the female laid an egg at a place, she moved to another place, paused for a short period, oviposited, and then moved to yet another place. In some cases, the female positioned the egg at the intersection of a whole bean and the substrate surface (Figure 2I). During egg laying, antennae were either moving freely in the air or they were tapping the food surface. None of these ovipositional behaviors were observed on or in the dish without food. A short representative video of *P. interpunctella* oviposition behavior is included in the Supplementary Information section.

Finally, *P. interpunctella* females often groomed their antennae and mouth parts after laying eggs and moving away from the food, in the manner described in Section 3.1.3.

There were significant differences in the mean numbers of eggs (±S.E.) laid in the food dish (63.3 ± 18.8), the empty dish (0.0 ± 0.0), and the arena proper (0.9 ± 0.6) (df = 2, χ^2^ = 1109.78, *p* < 0.01). The number of eggs laid in the food dish was significantly higher than those laid in the empty dish (*t* = 12.22, *p* < 0.01) or the arena proper (*t* = 11.13, *p* < 0.01). There were no differences in the number of eggs laid between the empty dish and the arena proper (*t* = −1.81, *p* = 0.11).

### 3.2. Study 2: Analysis of P. interpunctella Time Allocation Behavior during Oviposition

**Figure 4:**
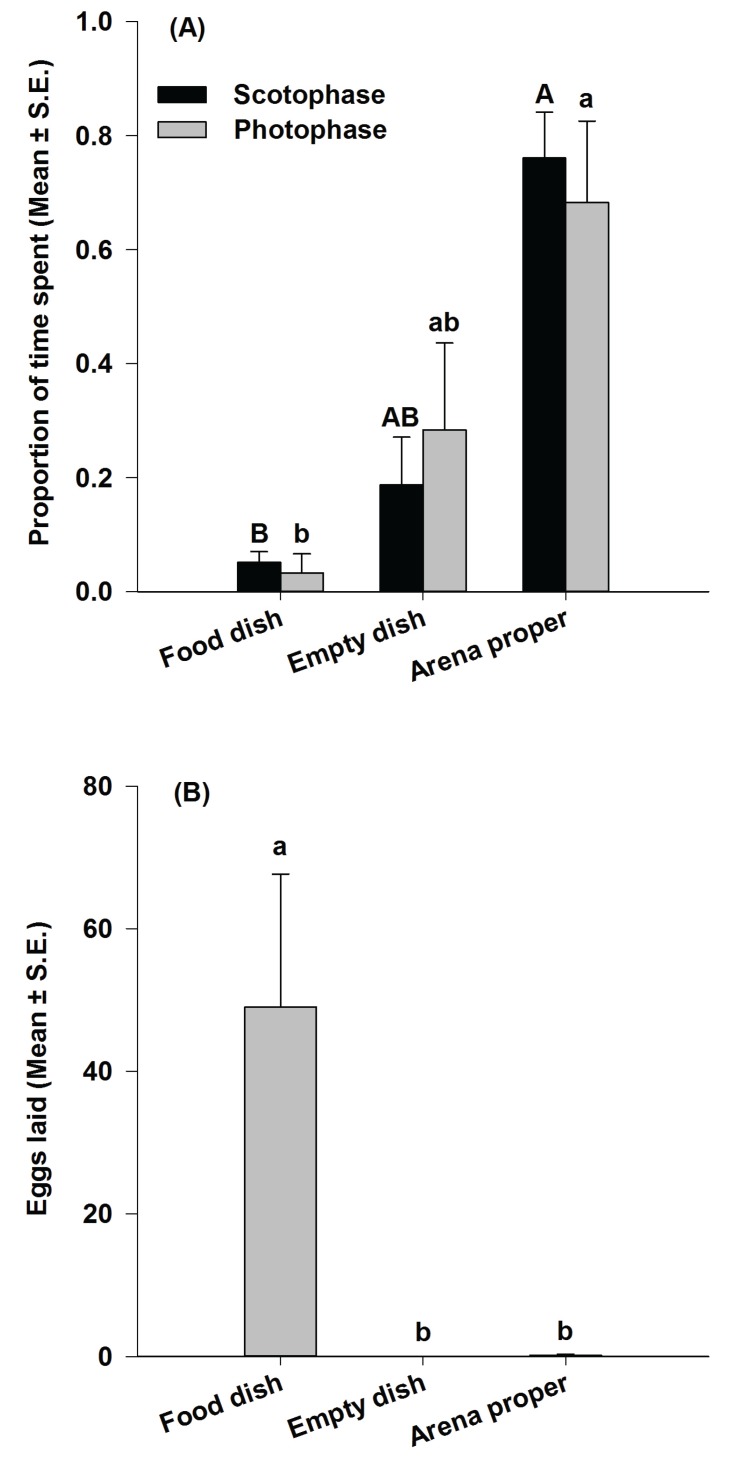
Proportion of time allocated during the scotophase and photophase periods in 48-h assays (**A**); and mean number of eggs laid (±S.E.) by *Plodia interpunctella* females (**B**) at the three oviposition sites in Study 2. Bars with the same uppercase or lowercase letter are not significantly different (*n* = 6; *p* < 0.01).

There was a significant association between the time spent by female moths during either the scotophase or the photophase and the site of oviposition (df = 2, χ^2^ = 209.32, *p* < 0.01). *Plodia interpunctella* females spent a significantly smaller proportion of time in the food dish *versus* the arena proper (*t* = −8.87, *p* < 0.01) during the dark phase. There were no differences in the proportion of time spent in the food dish and the empty dish (*t* = −1.46, *p* = 0.20) or the empty dish and the arena proper (*t* = −3.37, *p* = 0.02) during the dark phase (Figure 4A). Similarly, females spent a significantly smaller proportion of their photophase time in the food dish compared with the arena proper (*t* = −4.55, *p* < 0.01). There were no statistical differences between the proportion of time spent in the food dish *versus* the empty dish (*t* = −1.38, *p* = 0.23) or the empty dish *versus* the arena proper (*t* = −1.46, *p* = 0.20) during the photophase (Figure 4A). In other words, regardless of the phase, females spent a large proportion of their time in the arena proper, whereas time spent in the empty dish was intermediate between the arena proper and the food dish. No significant differences were observed between the proportion of photophase *versus* scotophase time spent by females in the three sites (*p* > 0.01).

The number of eggs laid in the food dish, the empty dish, and the arena proper was significantly different (df = 2, χ^2^ = 584.61, *p* < 0.01). A significantly greater number of eggs were laid in the food dish compared with either the empty dish (*t* = 7.62, *p* < 0.01) or the arena proper (*t* = 6.71, *p* < 0.01). No differences in egg counts were found between the empty dish and the arena proper (*t* = −1.00, *p* = 0.36) (Figure 4B).

**Figure 5:**
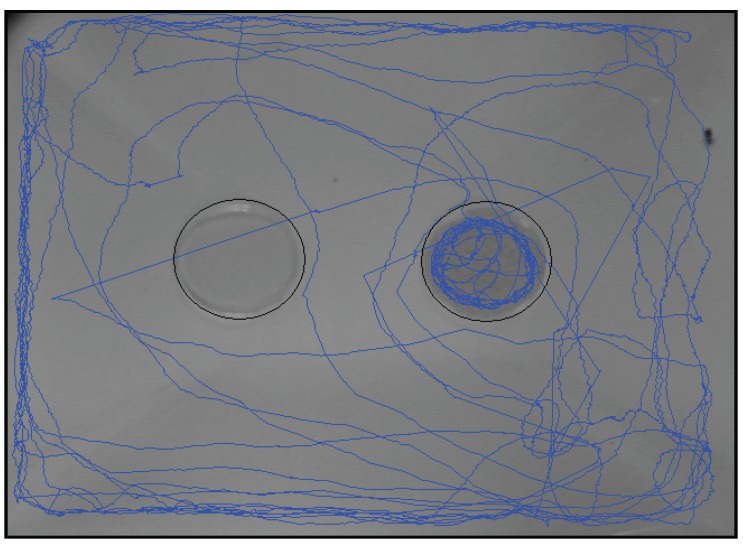
A representative image showing the movement pattern of a single *Plodia interpunctella* female for a 30-min period during the first 2 h of the scotophase. The experiment was a two-choice bioassay with an empty dish control (left); and a food dish (right) containing 2 g of cracked soybean.

A representative summary of moth movement during a 30-min recording period within the initial 2 h of a scotophase is presented in Figure 5. As opposed to moths being stationary and in their customary resting posture during the photophase irrespective of the oviposition site, active moths’ movements, particularly in the food dish, were observed primarily during the scotophase.

### 3.3. Study 3: Real-Time Analysis of the Visit Pattern of P. interpunctella Females Toward a Food Source

Gravid *P. interpunctella* females made several visits to a food source for oviposition during the scotophase. No significant differences in the number of visits by the female to the food dish were observed during the same 10-h periods on day 1 and day 2 (*t* = −1.88; *p* = 0.16; Table 1). Similarly, no significant differences in the time spent in the food dish were observed between day 1 and day 2 (*t* = −1.90; *p* = 0.15).

**Table 1:** Average number of visits and time spent (min ± S.E.) in the food dish by individual *Plodia interpunctella* females during two consecutive 10-h periods that each included an 8-h scotophase (*n* = 4).

Observation ^†^	Day 1	Day 2	Total
Number of visits	6.5 ± 1.2	11.5 ± 1.9	18.0 ± 1.6
Total time spent in food dish (min)	28.0 ± 9.3	53.9 ± 10.5	81.9 ± 11.9

^†^ No significant differences were observed between day 1 and day 2 (*p* < 0.01).

The moths were observed actively laying eggs during the initial few hours of the scotophase. The range of time spent per visit to the food dish, latency time between visits, and the number of eggs laid are shown in Table 2.

**Table 2:** Duration of single visits, visit latency, and number of eggs laid by *Plodia interpunctella* during two consecutive 10-h periods that each included an 8-h scotophase in Study 3.

Observations	
Duration of visit (range)	0.02–27.40 min
Duration of visit latency (range)	0.02–277.10 min
Average number of eggs laid in the dish ^†^	166.3 ± 70.2
Average total number of eggs ^†^	168.5 ± 70.8

^†^ Values are mean ± S.E. (*n* = 4).

**Figure 6:**
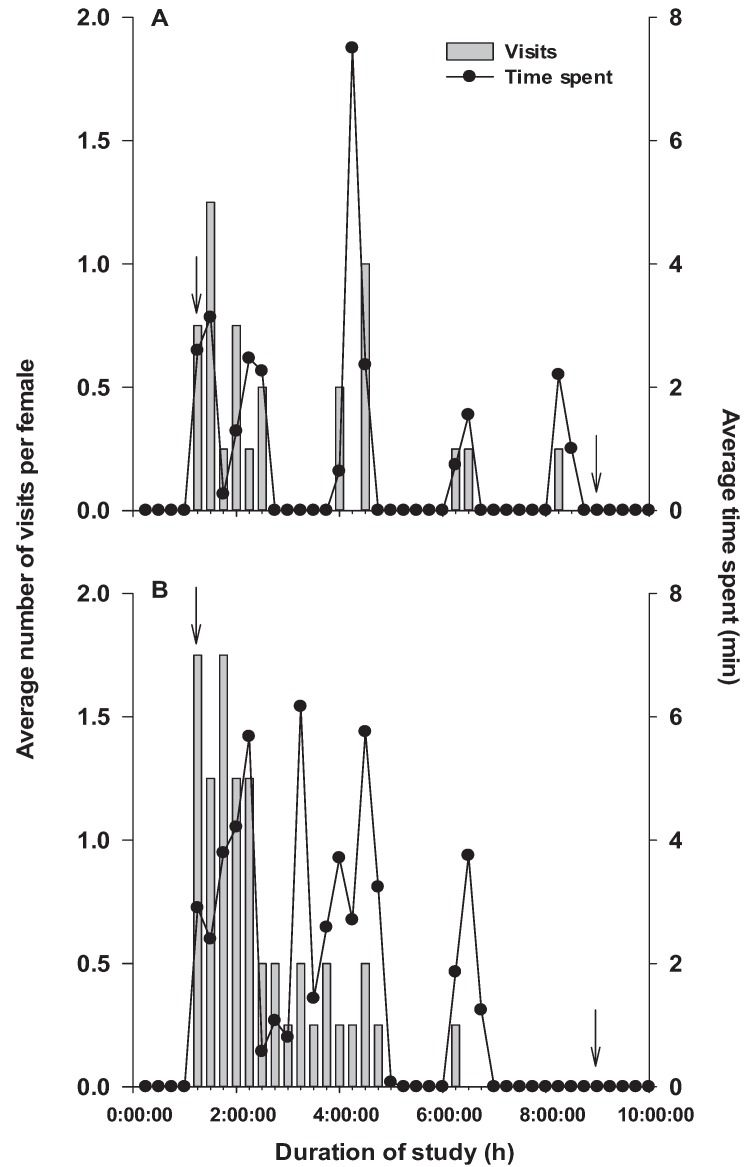
Mean number of visits (bars) and mean time spent on a food source (dots) by *Plodia interpunctella* during day 1 (**A**); and day 2 (**B**), recorded in Study 3. Each 10-h recording period for each day is divided into 40 consecutive 15-min intervals, and each bar or dot represents an average of observations for four females. Time interval between the downward-pointing arrows in each figure panel represents the duration of the scotophase. Standard errors for mean visits and mean time spent per 15-min interval were between 0–1.44 and 0–4.33 min, respectively.

Visits to the food dish during day 1 mostly occurred within 4 h of the beginning of the scotophase, after which a decline in the number of visits was observed (Figure 6A). Similarly, a greater number of visits occurred on day 2 during the initial 4 h of the scotophase than during the latter half of the scotophase (Figure 6B). Time of female activity in the food dish during day 1 varied among moths and mainly occurred within the first 4 h of the scotophase. Some foraging activity was also observed during the 6th and 8th hour of the scotophase on day 1 (Figure 6A). A similar pattern of moth activity in the food dish was observed during the scotophase of day 2, during which the moths predominantly spent time foraging in the food dish during the first 4 h of the scotophase (Figure 6B). Some activity was also observed during the 6th hour of the scotophase.

## 4. Discussion

Results of our laboratory investigations provide detailed information regarding the ovipositional behavior of female *P. interpunctella*. To summarize, gravid females: (1) sequentially exhibit antennal movement, grooming of antennae and mouth parts, walking/flying, abdomen bending and dragging, and finally oviposition on or near the food source after a shift from the photophase to the scotophase; (2) lay single eggs freely near the food or stick a single egg or a batch of 2–4 eggs on broken food material; (3) spend most of their time away from the food patch, irrespective of the presence or absence of light; (4) oviposit by making several visits to the food patch especially in the initial 3–4 h of the scotophase; and (5) spend varying amounts of time per visit, although the time spent generally declines in the later hours of the scotophase.

Pre-ovipositional behaviors exhibited by gravid *P. interpunctella* females, such as antennal movement, antennal rotation and antennal grooming, suggest attempts to perceive host volatiles or other stimuli. Similar behaviors have been observed in other species of moths such as the diamondback moth, *Plutella xylostella* (L.) (Lepidoptera: Plutellidae) [22] and the European small ermine moth, *Yponomeuta cagnagellus* (Hübner) [23]. Our study identified a brush-like structure, the tibial epiphysis, on the fore tibia of female *P. interpunctella*, that is apparently involved in grooming the antennae. Tibial epiphyses, such as those reported here for female *P. interpunctella*, are commonly found on the fore tibiae of several species of moths (including Pyralidae) and butterflies, and are used to clean the antennae [24,25,26]. The antennae of many species of moths are lined with uniporous and/or multiporous sensilla that perceive plant volatiles [27,28], and antennal cleaning presumably enhances their responsiveness to volatiles. In *P. interpunctella*, the antennae are lined with different types of sensillae that have chemosensory and gustatory functions and may thus be involved in host selection [29]. Deseo [30] showed that complete antennectomy of *P. interpunctella* females resulted in random egg laying, as opposed to directional egg laying on or near the food source by females with intact antennae*.*

*Plodia interpunctella* females were observed to use their fore tibiae to groom their proboscis and labial palpi in this work. Although the possible roles of the proboscis and labial palpi before or during oviposition were not investigated in our study, for several other species of the Lepidoptera, the proboscis, labial palpi, and tarsi play important roles during ovipositional host selection as they have contact chemoreceptors and mechanoreceptors [31,32,33]. Similarly, the ovipositor in several moth species is lined with mechanoreceptive and chemoreceptive sensillae [34,35,36,37], which hints that the ovipositor dragging behavior in *P. interpunctella* may have a sensory function. Ovipositor bending and dragging behavior is also observed in other species of moths such as the limabean pod borer, *Etiella zinckenella* (Treitschke) (Lepidoptera: Pyralidae) [38] and the tobacco budworm, *Heliothis virescens* (Fabricius) (Lepidoptera: Noctuidae) [39]. Nansen and Phillips [16] showed that when mated females were prevented from directly coming into contact with the food source, oviposition was significantly reduced. This study and our observations on oviposition behaviors suggest that the ovipositor in *P. interpunctella* might play an important role in host acceptance. An interesting observation that we made was that the female would climb on a whole bean and align its abdomen along the host surface and try to lay an egg near the junction between the host and the dish surface (Figure 2I). Similar behavior has been observed in the case of female angoumois grain moth, *Sitotroga cereallela* (Olivier) (Lepidoptera: Gelechiidae), which prefers to lay eggs on hosts that offer smaller crevices between the host seed and the substrate compared to those that offer bigger crevices [40]. Although the descriptions of pre- and post-oviposition behaviors in this work are based on moths commencing oviposition in a given time frame, we expect that the sequence of behaviors would be similar for mated moths that did not became active, or for those that became active but did not oviposit, in the stipulated time period.

Peak activities and oviposition patterns of many stored product insect species are influenced by photoperiod and time of the day [41,42,43], and our work confirms in more detail what was generally known about *P. interpunctella* oviposition and photoperiod [44,45]. Earlier studies showed that *P. interpunctella* movement and ovipositional rhythms are highly dependent on the photoperiod. Movements are erratic under continuous light as opposed to the more regular or routinely performed movements occurring under alternating light-dark periods [45]. Continuous light inhibits oviposition whereas, under continuous dark conditions, females show asynchronous oviposition patterns [44,45]. Alternating periods of light and low light intensity-dark elicit an optimal ovipositional response from the females, apparently as a result of the stimulus provided by the significant lowering of light intensity during the day-night transition [46].

We found that gravid females make several visits to a food source for oviposition that last from a few seconds to up to approximately 30 min with varying time latencies between visits, mostly within 4 h of the start of the scotophase. A similar type of oviposition behavior was observed in the unrelated *H. virescens*, whose females showed episodes of active oviposition combined with periods of rest or activity such as antennal movement and “wing buzzing” [39].

We elaborately described the oviposition behavior of *P. interpunctella* under idealized, simplistic laboratory conditions that would be subject to substantial ecological variation in nature. We chose soybean as ovipositional host material for our studies, but it is possible that other types of food elicit different quantitative ovipositional responses (e.g., visit patterns, *etc.*) from *P. interpunctella* females. Nevertheless, *P. interpunctella* has similar ovipositional preferences for a variety of foods [15,16], so we can expect that the general patterns of oviposition behavior will be similar to what we have described here, irrespective of the type of food. We used different amounts and particle sizes of soybean in our three studies rather than consistently using the same size of food pieces. These amounts and particle sizes were determined primarily based on preliminary experiments and ease of video recording. For example, in the first study, our preliminary investigations revealed that (1) *P. interpunctella* females tend to climb the whole seed and oviposit near its base; and (2) females stick their eggs on the cracked seeds. Therefore, we used both whole and cracked seeds, but in the case of automated recording of *P. interpunctella* movements using a software program, only cracked soybean was used because whole soybeans would have resulted in shaded/dark regions that would have interfered with the recording of the insect’s movements during the scotophase. Our work provided new information on female behaviors with regard to orientation to food and placement of eggs as well as detailed observations due to the efficiency of the video tracking software, which might not have been possible with other food structures.

Our studies provide important insights into the ovipositional behavior and ecology of *P. interpunctella* that can inform and facilitate a variety of future research. For example, more research is needed to fully understand the sensory processes and behavioral mechanisms involved in host selection by *P. interpunctella* females. Few studies have investigated the morphology, distribution and diversity of sensilla on the female sensory apparatus of *P. interpunctella* [29]. Furthermore, a detailed understanding of individual-level behaviors such as oviposition and feeding could help devise simple, yet highly effective pest control methods for stored product insects. For instance, rolling bean containers was found to be highly effective in reducing populations of the common bean weevil, *Acanthoscelides obtectus* (Say) (Coleoptera: Chrysomelidae), based on the fact that *A. obtectus* larvae need adjacent hard surfaces, such as those formed by other beans or a container, to successfully bore into the beans [47]. A similar management approach may be possible with *P. interpunctella* in places where grains, seeds, or their mixtures (e.g., bird seed) are stored in small containers or bags. As *P. interpunctella* females lay eggs loosely or sometimes stick them to broken food pieces, a moderate disturbance such as shaking of grain or seed containers or bags could cause serious egg mortality, potentially reducing populations of this insect. However, more research is needed to quantify the efficacy of this method. Finally, the knowledge concerning the influence of light-dark conditions on *P. interpunctella* may be used for managing moth populations, for instance, by applying insecticides during periods of maximal activity of ovipositing moths, *i.e.*, early scotophase, in storage warehouses.

## 5. Conclusions

The controlled observations of single gravid *P. interpunctella* females’ orientation towards soybeans in our studies provide detailed information on oviposition behavior for this important pest of stored products. Key behaviors leading up to and immediately following oviposition were recorded by videography, and specific behaviors were further documented with high-quality line drawings. Our detailed time budget analysis provides insights into the oviposition behavior of *P. interpunctella*, namely the characteristic behaviors displayed, the time allocated, and the visits made during foraging. Females were essentially inactive during the photophase on any given day, but oviposition commenced on a localized food source generally within 15 min of the start of the scotophase, with all eggs for a given day apparently being laid within the first 4 h of the scotophase. An understanding of the pattern and timing of egg deposition gained from this work suggests that simple changes or disturbances to the physical storage environment could limit infestation by *P. interpunctella* via unsuccessful oviposition and/or egg mortality.

## Supplementary Files

Supplementary File 1
